# Public Policies in the informational era: a compared perspective in the context of pandemic in two Lusophone countries

**DOI:** 10.31744/einstein_journal/2022ED6252

**Published:** 2022-01-27

**Authors:** Thiago Gonçalves dos Santos Martins, Flavia de Souza Rangel, Luís Guilherme Arneiro Mendes, Rufino Silva

**Affiliations:** 1 Universidade de Coimbra Coimbra Portugal Universidade de Coimbra, Coimbra, Portugal.; 2 Universidade Estácio de Sá Rio de Janeiro RJ Brazil Universidade Estácio de Sá, Rio de Janeiro, RJ, Brazil.

In the information age, technological developments in the area of big data and artificial intelligence have also been used in the development of new governmental Public Policies. These technologies must consider the implications of their decisions, which have particularities according to each administrative state.^(1)^ In this new era, governments have increasingly used the citizen in co-production and information. For this reason, to understand the citizen’s profile becomes a fundamental part in the planning and execution of Public Policy strategies.^(2)^

The pandemic caused by the coronavirus 2019 disease (COVID-19) has become a global health crisis, affecting countries differently and requiring urgent Public Policy measures to prevent virus transmission and increased mortality rate. Approximately half of all infections occur before the symptomatic phase. For this reason, isolation only after the symptomatic phase is not enough to reduce the transmission rate. The use of artificial intelligence in the development of contact tracing algorithms using cell phone technology has proven to be an important tool in limiting the transmission of the disease.^(3)^

The literature was searched to understand the epidemiological situation of two Lusophone countries (Brazil and Portugal) up to September 19, 2020 and to compare two algorithms applied in Public Policies used in these two countries, which provide epidemiological data in real time, and allowed the adjustment of Public Policies.

## BRAZIL

**Figure 1:**
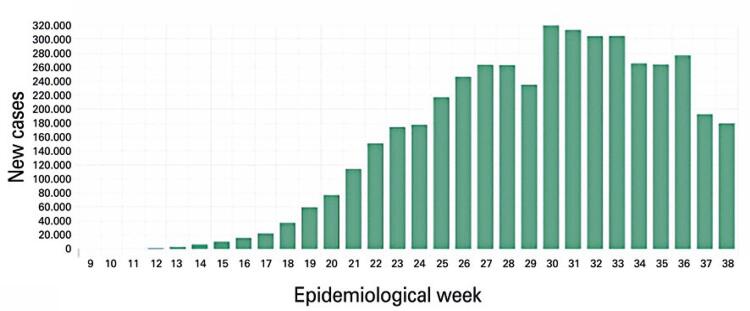
Total cases of COVID-19 in Brazil in September 19, 2020

Source: Instituto D´Or de Pesquisa e Ensino; Zoox Smart. DataDados do bem [Aplicativo]. [citado 2020 Set 19]. Disonível em: https://dadosdobem.com.br/termos-de-uso/^(5)^

**Figure 2:**
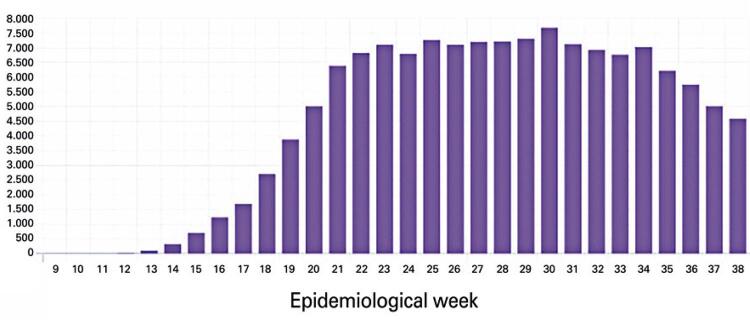
Total deaths from COVID-19 in Brazil by September 19, 2020

Source: Instituto D´Or de Pesquisa e Ensino; Zoox Smart. DataDados do bem [Aplicativo]. [citado 2020 Set 19]. Disonível em: https://dadosdobem.com.br/termos-de-uso/^(5)^

In September 19, 2020, Brazil had a total of 4,495,183 cases and 135,793 deaths (Figures 1 and 2). The country began to have a reduction in deaths from the 35th epidemiological week (August 2020). The reduction in the number of documented cases began to occur from the 37th epidemiological week (September 2020).^(4)^

The Brazilian application selected for analysis was “*Dados do bem*/Good data”, developed to analyze the evolution of immunity of the Brazilian population, which uses artificial intelligence algorithms. This application was designed by the *Instituto D’Or de Pesquisa e Ensino* (IDOR) and Zoox Smart Data. The application depends on the collaboration of the population to provide the data.

In this context, participants must download the application on their cell phone, which does not diagnose the disease, but rather performs an assessment of clinical and epidemiological signs. Participants make a self-assessment of their symptoms, answers data about COVID-19 symptoms, and his health history. Subsequently, the application locates possible severe acute respiratory syndrome coronavirus 2 (SARS-CoV-2) infections. Based on generated data, participants are randomly drawn by the algorithm for in-person testing. Therefore, allowing data on the spread of COVID-19 to be generated.

If the data completed during the self-assessment of symptoms indicate the possibility of contamination, the participant receives a code on the phone with date and time to go to a testing station. If the patient has a positive result, there is a request to indicate people with whom he or she had contact to perform the self-assessment through the application (Table 1).

**Table 1:** Comparison between two contact tracing applications used in Brazil and Portugal

Algorithms	Good data	STAYAWAY COVID
Participation of private initiative	Yes	Yes
Sharing of data for Public Policies	Yes	Yes
Running out of national territory	Brazil only	European community (Portugal)
Running in IOS/Android	Yes	Yes
Advise to participants	No	Yes
Data inclusion	The individual himself complete the questionnaire (questions about symptoms) after downloading the application	After testing positive for COVID-19, the individual receives a code from their physician and, if desired, he/she enters the code into the App. Everyone who had contact with the patient within the last 14 days will be notified and they can seek health care (optional)
Tracking form	Individual is drawn to take the exam, after complete the questionnaire, and indicates people with whom he or she had contact to do the self-assessment through the application	Bluetooth on (digital tracking without revealing the identity of the infected individuals)
Protection of individuals data	Yes	Yes
Request for laboratory exams	Yes (after complete the questionnaire, by drawing - sample testing). This is not mandatory	Yes (after contact with infected patient - digital scanning). Not required
Disposal of data	Individuals agree to voluntary involvement in the study by downloading the App. No data are discarted	Disposal of stored data after the pandemic

## PORTUGAL

**Figure 3:**
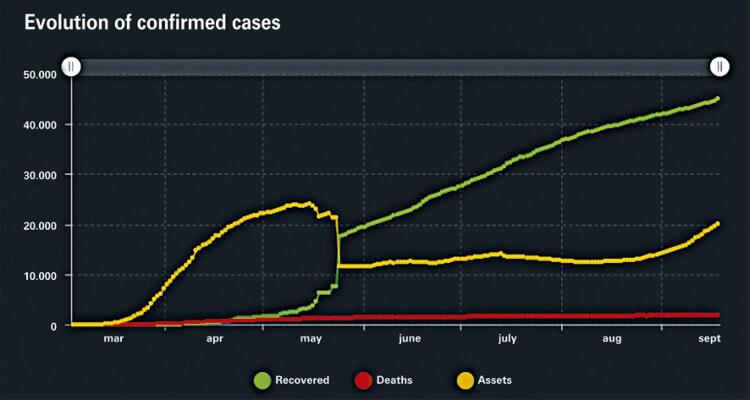
Total cases and deaths in Portugal until September 19, 2020. Data that can be optimized and updated in real time using the application

Source: Portugal. Direção Geral da Saúde. COVID-19. Ponto de situação atual em Portugal. Portugal: Direção Geral da Saúde; 2020 [citado 2021 Jan 11]. Disponível em: https://covid19.min-saude.pt/ponto-de-situacao-atual-em-portugal/^(6)^

Portugal had until September 19, 2020, a total of 67,176 confirmed cases and 1,894 deaths since the beginning of the pandemic (Figure 3). There was a relative control of the pandemic, with a stable number of deaths and reduction of confirmed cases after the mid-May 2020.^(6)^

The algorithm chosen for analysis was STAYAWAY COVID, developed based on Public Policies already taken within the European community to fight the COVID-19 pandemic. This application was developed by the Decentralized Privacy-Preserving Proximity Tracing” (DP-3T or dp3t, - Decentralized Privacy-Preserving Proximity Tracing) initiative and by the Institute for Systems and Computer Engineering, Technology and Science (INESC TEC). This initiative assumed the responsibility of the development of a complete system that could be implemented to support in Public Policies to combat COVID-19.

In the application, the participant must enter a code that confirms his or her diagnosis of COVID-19, and the algorithm itself send a message to possible contactors, without revealing the identity of the infected person. The algorithm defines a possible contact person as someone who has been within 2 meters of an infected individual for at least 15 minutes. The insertion of data in the application is done voluntarily, and demonstrates the use of technology in co-participation with the population in the development of Public Policies to fight COVID-19.

The algorithm requires the Bluetooth of the cell phone to be turned on in order to function properly. In order to guarantee the confidentiality of the data, only the cell phone user receives the information about being a possible contact person. The whole system will be discontinued when the end of the pandemic is declared in Portugal. STAYAWAY COVID aims to cross-reference data with the largest number of European and non-European COVID-19 digital tracking initiatives that have similar tracking algorithms. The application works in any country of the European community (Table 1).

It is important to highlight that technological innovation with the application of artificial intelligence aims to reduce public expenses, which is high using the traditional models, which require more employees and inputs. On the other hand, data privacy is challenging, since location data are highly confidential.^(7)^

Note that the application adopted by Portugal (digital contact tracing) uses electronic information to identify exposures to infections, unlike what happens in Brazil. It has the potential to address the limitations of traditional contact tracing, such as scalability, notification delays, recall errors, and contact identification in public spaces. Notifications can be sent quickly and automatically to potential contacts, recommending isolation until medical confirmation of the diagnosis.

Both applications presented depend on the coparticipation of the population for effective results. If a significant percentage of the population does not participate due to fears including privacy issues relate to the implemented Public Policy may have limited impact on controlling the pandemic. Another constraint to the operation of both Apps is the population’s access to smartphones and the internet. In Brazil, in the year 2019, there was a estimation of 107.29 cell phones/per 100 inhabitants, and in Portugal, in 2018, there was a estimation of 120.2 cell phones/per 100 inhabitants. Thus, for these two countries, the use of App. on cell phones may be a good option, since they have more devices than inhabitants.^(8)^ However, it should be remembered that, regardless of the number of inhabitants who have a cell phone or not, there are other structural problems in countries like Brazil, which include digital illiteracy, and regions of the country where Human Development Index does not reflect the statistics presented.

A further expected difficulty with the algorithms is that some fraction of users will not report their confirmed diagnosis, either due to negligence or privacy issues. The Apps are only able to identify contacts when the infected and exposed individuals have their phones close to each other and when both individuals are using interconnected Apps. All contact tracking Apps have reduced effectiveness in communities where smartphone ownership is limited, as well as when individuals share smartphones, or where people are unable or unwilling to use an App.

Local privacy and data protection laws must be respected. This is made possible with encryption of all personal data, user consent for data storage and use, restrictions on data use outside of public health responses to COVID-19, automatic deletion of data, and the option to delete data at any time. The deletion of data, while ensuring user privacy, limits retrospective studies of the data that seek improved effectiveness of algorithms. On the other hand, the technology used in Brazil does not adopt digital tracking, but allows the storage of data collected by the voluntary inclusion of participants, in an unidentified form, for future studies, as expressly provided in the terms of use of the application of Good Data.^(5)^

The use of an App should be voluntary, and users have the option to pause contact localization, in order to further protect privacy and allow healthcare professionals to disable monitoring when using appropriate precautions, and avoid incorrect notifications regarding digital tracking.

It should also be noted that the algorithms do not cover all possible routes of transmission between individuals, as viruses can be present on surfaces, and direct contact with other people is not necessary.

It is noted that for applications that use bluetooth, such as STAYAWAY COVID used by Portugal, present problems regarding the use of signal strength to determine the distance between devices: the bluetooth signal strength depends on the hardware, present fluctuations and it is attenuated when people and objects are between the devices. However, they also have advantages, since they only detect signals from other users who are within a few meters (distance required for transmission).

It should be noted that digital tracking technology allows the users’ application to capture a temporary identification of all nearby users and use this encrypted information for the tracking of possible contactors. Once the user receives the diagnosis, they can voluntarily share their data with a central server, which sends a notification to all applications that had a temporary ID generated near the infected user’s application. In this way the user receives this information and has their data protected. The user can then go to the nearest health care facility to conduct laboratory tests.^(9)^

It is also possible to identify false exposures with health care professionals who, despite being close to infected individuals, may not have become infected because they wear personal protective equipment. However, they should not be excluded, as they represent an important fraction of the possible cases, thus recommending a differentiated control of these professionals, to avoid unnecessary quarantine, and hinder the provision of adequate service to society.^(10)^

It is also emphasized that the limitation of the application operation in other territories makes it difficult to use them in border cities, which have separate public health jurisdictions and high transit of people of different nationalities. Coordination between public health agencies could optimize the use of the algorithms in these locations.

Finally, it is identified that the applications presented the need of longitudinal studies to prove their theoretical effectiveness. Of note is that, whether the large-scale use of these Apps leads to a reduction in other preventive measures or reduced cooperation in traditional contact tracing, their impact should be small and potentially negative.

## References

[1] Barth TJ, Arnold E. Artificial intelligence and administrative discretion: implications for public administration. Am Rev Public Adm. 1999;29(4):332-51.

[2] Tinholt D, Carrara W, Linden N. Unleashing the potential of artificial intelligence in the public sector. Capgemini Consulting; 2017 [cited 2020 Sep 24]. Available from: https://www.capgemini.com/consulting/wp-content/uploads/sites/30/2017/10/ai-in-public-sector.pdf

[3] Ferretti L, Wymant C, Kendall M, Zhao L, Nurtay A, Abeler-Dörner L, et al. Quantifying SARS-CoV-2 transmission suggests epidemic control with digital contact tracing. Science. 2020;368(6491):eabb6936. 10.1126/science.abb6936 PMC7164555 32234805

[4] Brasil. Ministério da Saúde. Coronavírus Brasil. Painel Coronavírus. Versão 2.0. Brasília (DF): Ministério da Saúde; 2020 [citado 2020 Set 23]. Disponível em: https://covid.saude.gov.br/

[5] Instituto D´Or de Pesquisa e Ensino; Zoox Smart. DataDados do bem [Aplicativo]. [citado 2020 Set 19]. Disonível em: https://dadosdobem.com.br/termos-de-uso/

[6] Portugal. Direção Geral da Saúde. COVID-19. Ponto de situação atual em Portugal. Portugal: Direção Geral da Saúde; 2020 [citado 2021 Jan 11]. Disponível em: https://covid19.min-saude.pt/ponto-de-situacao-atual-em-portugal/

[7] Farrahi K, Emonet R, Cebrian M. Epidemic contact tracing via communication traces. PLoS One. 2014;9(5):e95133. 10.1371/journal.pone.0095133 PMC4006791 24787614

[8] Teleco Inteligência em Telecomunicações. Estatísticas do Brasil – Geral. [citado 2020 Set 19]. Disponível em: https://www.teleco.com.br/estatis.asp

[9] Li G, Geng E, Ye Z, Xu Y, Lin J, Pang Y. Indoor Positioning Algorithm Based on the Improved RSSI Distance Model. Sensors (Basel). 2018;18(9):2820. 10.3390/s18092820 PMC6165244 30150521

[10] CDC COVID-19 Response Team. Characteristics of Health Care Personnel with COVID-19 - United States, February 12-April 9, 2020. MMWR Morb Mortal Wkly Rep. 2020;69(15):477-81. 10.15585/mmwr.mm6915e6 PMC7755055 32298247

